# Rho-kinase inhibitor Y-27632 and hypoxia synergistically enhance chondrocytic phenotype and modify S100 protein profiles in human chondrosarcoma cells

**DOI:** 10.1038/s41598-017-03958-5

**Published:** 2017-06-16

**Authors:** Juha Piltti, Joakim Bygdell, Cecilia Fernández-Echevarría, Daniel Marcellino, Mikko J. Lammi

**Affiliations:** 1 Department of Integrative Medical Biology, Umeå University, Linnaeus väg 9, 90187 Umeå, Sweden; 2 Computational Life Science Cluster (CLiC), Department of Chemistry, Umeå University, Linnaeus väg 10, 90187 Umeå, Sweden; 3 School of Public Health, Health Science Center of Xi’an Jiaotong University, Key Laboratory of Trace Elements and Endemic Diseases, National Health and Family Planning Commission, Xi’an, China

## Abstract

Articular chondrocytes are slowly dividing cells that tend to lose their cell type-specific phenotype and ability to produce structurally and functionally correct cartilage tissue when cultured. Thus, culture conditions, which enhance the maintenance of chondrocyte phenotype would be very useful for cartilage research. Here we show that Rho-kinase inhibition by Y-27632 under hypoxic conditions efficiently maintains and even enhances chondrocyte-specific extracellular matrix production by chondrocytic cells. The effects of long-term Y-27632 exposure to human chondrosarcoma 2/8 cell phenotype maintenance and extracellular matrix production were studied at normoxia and at a 5% low oxygen atmosphere. Y-27632 treatment at normoxia induced ACAN and COL2A1 gene up-regulation and a minor increase of sulfated glycosaminoglycans (sGAGs), while type II collagen expression was not significantly up-regulated. A further increase in expression of ACAN and COL2A1 was achieved with Y-27632 treatment and hypoxia. The production of sGAGs increased by 65.8%, and ELISA analysis revealed a 6-fold up-regulation of type II collagen. Y-27632 also induced the up-regulation of S100-A1 and S100-B proteins and modified the expression of several other S100 protein family members, such as S100-A4, S100-A6, S100-A13 and S100-A16. The up-regulation of S100-A1 and S100-B proteins is suggested to enhance the chondrocytic phenotype of these cells.

## Introduction

Articular cartilage is a poorly regenerating tissue, and there is need for effective repair techniques to treat damaged cartilage tissues. Many methods are used to treat damaged cartilage, but existing biological therapies do not provide the formation of native-type hyaline cartilage repair tissues. Current cell therapies are predominantly based on the use of autologous chondrocytes, while the feasibility of stem cell-based therapies is actively studied. Regardless of the cell source, differentiated chondrocytes have the tendency to lose their phenotype and ability to produce cartilage-specific extracellular matrix over time^1^. Many therapeutic applications require *in vitro* culturing of chondrocytes to expand the amount of cells, thus, the dedifferentiation of chondrocytes *in vitro* is a challenge. In addition, some possible weaknesses of the current chondrogenic differentiation processes have been recognized. Thereby, improvements are needed to promote chondrocyte differentiation and to achieve protein expressions of adult chondrocytes and chondrogenically differentiated stem cells^2^. The inhibition of RhoA/ROCK signaling induces chondrogenic markers, such as the activity of transcription factor SOX9 and expression of transcripts of ACAN and COL2A1 genes^3^. Rho-kinase inhibitor Y-27632 has previously been shown to maintain the differentiated phenotype of articular chondrocytes without producing harmful effects, *i.e*., terminal hypertrophic differentiation or reductions in cell number^4^. Moreover, ROCK inhibition increased ACAN gene expression and down-regulated gene expression of the catabolic matrix metalloproteinase-3 (MMP-3) in a balanced way^5^.

The oxygen strain of healthy avascular cartilage tissue is estimated to be 5%. The cellular microenvironment is known to regulate cell phenotype. Furthermore, both microenvironment and paracrine factors can regulate extracellular matrix production and chondrogenic differentiation of mesenchymal stem cells^6^. This study was performed to investigate the specific responses to prolonged Rho-kinase inhibition at normoxia and at a 5% low oxygen atmosphere in human chondrosarcoma-2/8 cells (HCS-2/8). A proteomic-based analysis was performed to reveal which protein responses are produced by Y-27632 at specific oxygen atmospheres. An advantage of using proteomic methodology is its ability to confirm known and to reveal potentially novel protein markers for chondrogenesis and differentiation status of cells. Previously, proteomic-based analyses have been used to define protein profiles of articular chondrocytes and changes related to chondrogenic differentiation and osteoarthritis^7–12^. That information is used as a reference to evaluate the proteomic screening results of this study. The human HCS-2/8 cell line has a prominent aggrecan and type II collagen production, and it shares most features of normal chondrocytes^13, 14^.

## Results

### Time-lapse imaging

**Figure 1:**
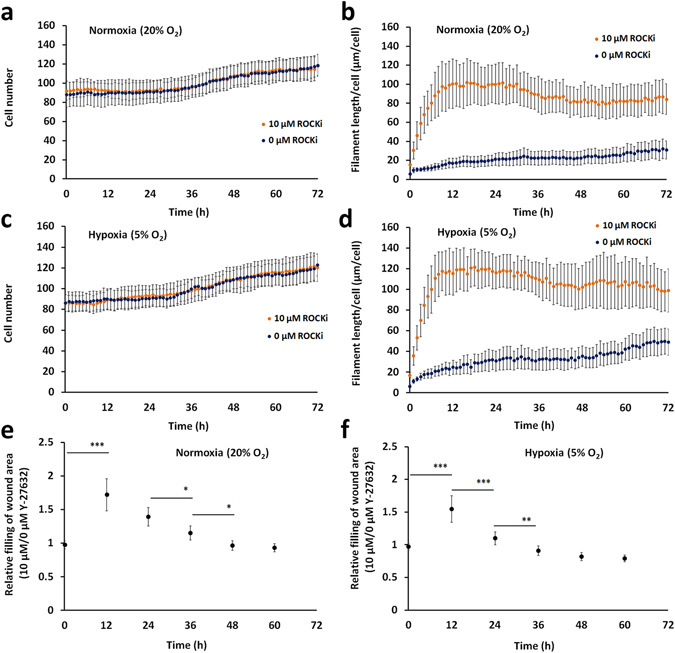
Cell-IQ time-lapse imaging assay. The cell number and the average filament length of control and 10 µM Y-27632-treated cells at normoxia (**a** and **b**). The cell number and the average filament length of control and 10 µM Y-27632-treated cells at hypoxia (**c** and **d**). The relative filling of the wound area of Y-27632-treated cells *vs*. controls at normoxia (**e**) and at hypoxia (**f**). Rho-kinase inhibitor induced a short transient increase in filling rates at both oxygen atmospheres. Cell monitoring was repeated three times with at least two replicates and the scratch wound assay was performed three times each with six replicates.

A three day exposure to 10 µM Y-27632 did not affect the cellular proliferation of HCS-2/8 cells, rather this treatment inhibited the depolymerization of filamentous actin (Fig. 1, Supplementary Fig. 1), similar to finding observed in human fibroblasts^15^. The formation of protrusions during Y-27632 treatment complicates the estimation of cellular phenotype. The appearance of long cellular extensions indicates that HCS-2/8 cells responded to Y-27632 exposure. Using a scratch wound migration assay, the kinetics of HCS-2/8 cell migration were slightly increased during the first 12 hours after the addition of Y-27632 at both oxygen atmospheres (Fig. 1, Supplementary Fig. 2). The wound area of Y-27632-treated cultures was, on average, 61% ± 24% smaller compared to controls at normoxia, and 55% ± 20% smaller than control scratch wounds at 5% oxygen at the 12-hour time point. After this time point, the differences between wound areas disappeared. After 60 hours at normoxia, the Y-27632-treated cultures reached 75.4% ± 3.5% and controls reached 78.9% ± 3.9% confluency of the total area of the monitored grid. Correspondingly at 5% oxygen atmosphere, Y-27632-treated cells achieved 66.7% ± 2.9% and controls 75.1% ± 3.5% of confluency. Thereby, statistically significant differences between the stages of confluency were found in Y-27632-treated and control cells only under hypoxic conditions.

### Proteomic analyses

**Figure 2:**
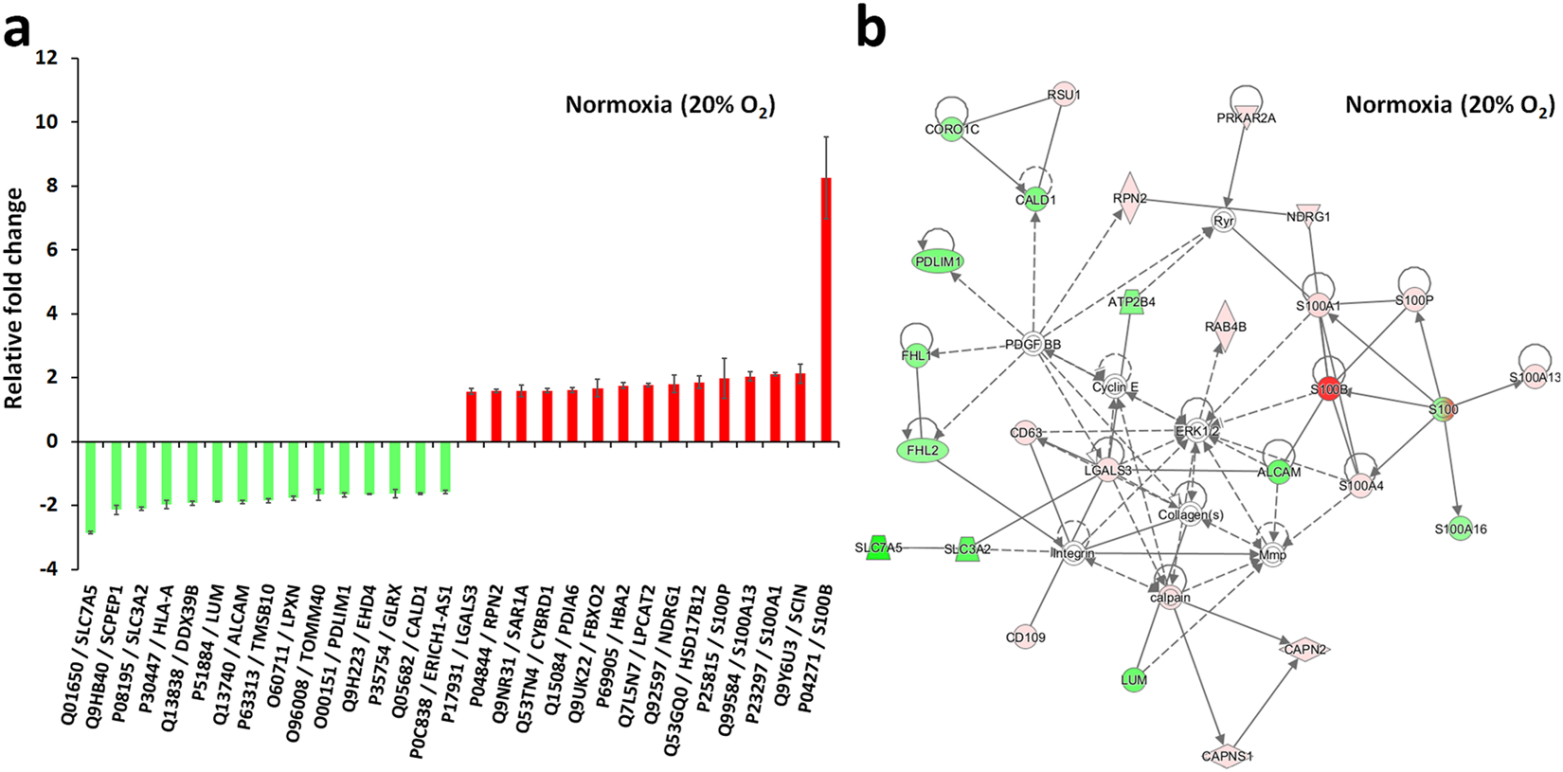
The fifteen most up-regulated and down-regulated proteins produced by a 28-day exposure to 10 µM of the Rho-kinase inhibitor Y-27632 (**a**). An IPA^®^ protein pathway analysis suggested a molecular network with the highest number of focus molecules (focus molecules 26, score 59) (**b**). This network was predicted to connect to organismal abnormalities and cancer. Error bars indicate 95% confidence intervals (*n* = 3).

**Figure 3:**
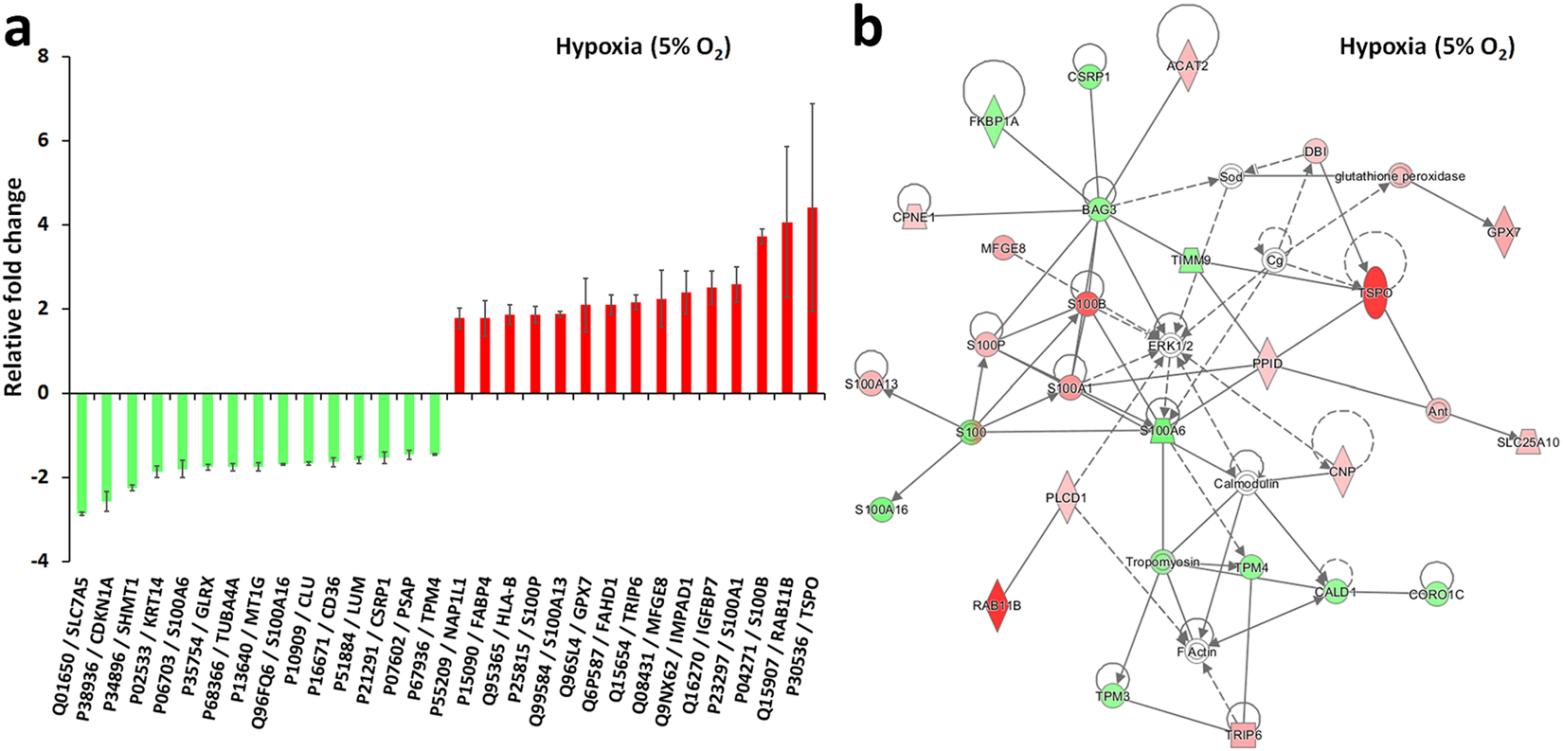
The fifteen most up-regulated and down-regulated proteins produced by a 28-day exposure to 10 µM Y-27632 at 5% oxygen atmosphere (**a**). An IPA^®^ protein pathway analysis suggested a molecular network with the highest number of focus molecules (focus molecules 26, score 59) (**b**). The network was connected to both molecular transport and to cell death and survival. The IPA^®^ analysis was not able to interpret at the network level an increase in extracellular matrix production. An additional secretome analysis could improve the power of the pathway analysis in the context of an extracellular matrix study. Error bars indicate 95% confidence intervals (*n* = 3).

A quantitative analysis of the proteome revealed numerous protein responses produced by long-lasting 10 µM Y-27632 treatment both at normoxia and at 5% low oxygen atmospheres. In total, Y-27632 treatment at normoxia changed cellular protein levels of 109 proteins using a minimum fold change set to ±1.3 (Fig. 2a, Supplementary Table 2). Correspondingly, Y-27632 treatment at hypoxia produced changes in 101 proteins (Fig. 3a, Supplementary Table 3).

At normoxia, long-lasting 10 µM Y-27632 exposure produced 62 up- and 47 down-regulated proteins in HCS-2/8 cells. An IPA^®^ protein pathway analysis suggested four major activated protein networks. The largest target protein network had relations to cancer, organismal injury and abnormalities (score 59, Fig. 2b). All cancer-related findings potentially associate with the chondrosarcoma model used in this study. The second and the third potential protein networks (scores 44 and 42) had connections to cell survival and death, and may also partly relate to the cell line used. The fourth network (score 41) was related to amino acid metabolism, hematological system development and function, and tissue development. A detailed inspection of the networks suggested a decrease in binding of cells (activation z-score 2.418, p-value of overlap 9.62E-03), an increase of apoptosis (activation z-score 1.645, p-value of overlap 1.12E-03), and an increase in proliferation of cancer cells (activation z-score 1.588, p-value of overlap 6.31E-03). However, the last presumption is controversial since the proliferation of prostate cancer lines was predicted to decrease (activation z-score -1.387, p-value of overlap 9.29E-03).

Long-lasting 10 µM Y-27632 treatment produced several S100 protein family member-related changes in the HCS-2/8 proteome. Protein S100-B had the highest up-regulation, which was multifold in comparison to other up-regulated S100 proteins (Fig. 2a). In addition, S100-A1, S100-A13, S100-P and S100-A4 were also up-regulated at levels that were at least 1.5 times or higher. In contrast, the protein S100-A16 was down-regulated by 1.3 fold.

At 5% hypoxia, 10 µM Y-27632 treatment produced 64 up- and 37 down-regulations of proteins. An IPA^®^ analysis revealed the potential connections to molecular transport and to cell death and survival (26 target molecules, score 59, Fig. 3b). The second largest network (score 39) was related to cell cycle, cellular growth and proliferation, and to tissue morphology. The third network (score 34) had similarities with protein networks modified by Y-27632 at normoxia, networks linked to cancer, organismal injury and abnormalities. A detailed inspection of context-dependent predicted network categories anticipated a potential decrease in cell death of connective tissue cells (activation z-score -2.864, p-value of overlap 5.24E-05), an increase of cellular protrusions (activation z-score 1.406, p-value of overlap 1.27E-03), and formation of filaments (activation z-score 1.210, p-value of overlap 4.32E-03). IPA^®^ pathway analysis also interpreted that Y-27632 treatment at hypoxia had certain influences on lipid metabolism in HCS-2/8 cells. An efflux of cholesterol (activation z-score -1.969, p-value of overlap 1.71E-03) and the transport of cholesterol (activation z-score -1.501, p-value of overlap 3.53E-03) were predicted to decrease. Y-27632 exposure at hypoxia did not induce cell migration (activation z-score 0.040). The IPA^®^ analysis also recognized hypoxia-induced VEGFA activation (activation z-score 1.491, p-value of overlap 7.40E-06), which is related to hypoxia-inducible factor (HIF) pathway.

**Figure 4:**
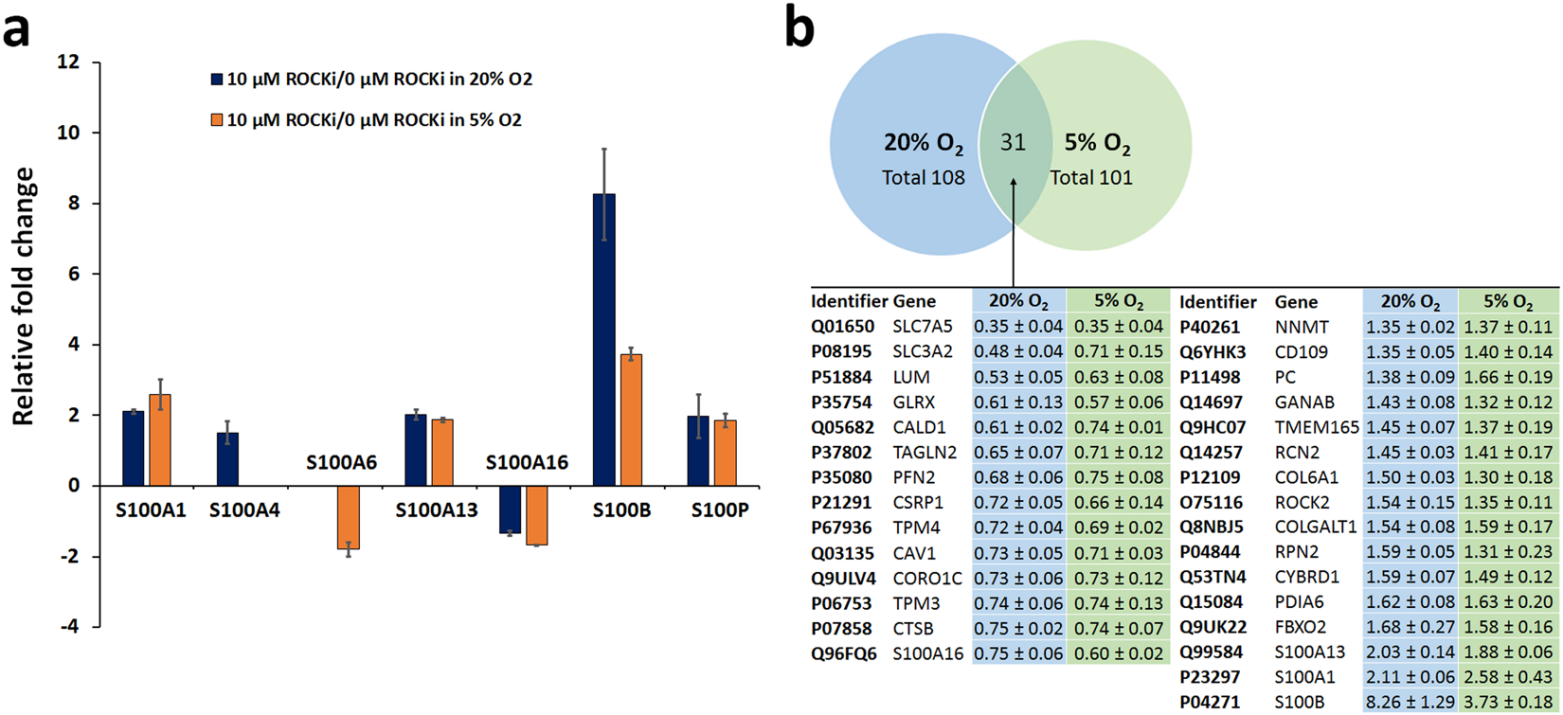
Long-lasting Y-27632 treatment produced several S100 protein fold changes in HCS-2/8 cells (**a**). Y-27632 increased S100-B expression the most. The second highest up-regulated S100 protein was S100-A1 although, S100-A13 and S100-P were relatively close to this increase. Y-27632 down-regulated S100-A16 expression and both S100-A4 and S100-A6 proteins had oxygen-dependent regulation. The Venn diagram depicts the internecine protein fold changes produced by the Rho-kinase inhibitor Y-27632 at normoxia and at hypoxia (**b**). A long-lasting 10 µM Y-27632 exposure induced 31 oxygen-independent protein responses in human HCS-2/8 cells. The internecine fold changes are represented only for proteins that reached an ANOVA *p*-value equal to or less than 0.05.

10 µM Y-27632 produced several changes to S100 protein family members also at hypoxia (Fig. 4a). Similar to normoxic conditions, protein S100-B was the most up-regulated member of S100 protein family. Although a Y-27632-induced up-regulation of S100-B was less pronounced at hypoxia, it was still 3.7 times higher than controls. At hypoxia, Y-27632 up-regulated S100-A1, S100-A13, S100-P proteins, while S100-A6 and S100-A16 proteins were down-regulated. The Y-27632-induced up-regulation of S100-A4 at normoxia was not observed at hypoxia and the S100-A6 down-regulation was observed only at hypoxic conditions.

Noteworthy, 10 µM of Y-27632 at hypoxia produced changes in sulfation-related enzymes. Two cytosol-located enzymes, namely bifunctional 3′-phosphoadenosine 5′-phosphosulfate synthases 1 and 2 (PAPSS1 and PAPSS2, respectively), were found to have elevated protein levels. These enzymes catalyze the biosynthesis of 3′-phosphoadenosine 5′-phosphosulfate (PAPS) which serves as a universal sulfonate donor compound for all sulfotransferase reactions^16^. At the first step, PAPSS1 takes part in conversion of sulfate to adenosine 5′-phosphosulfate, and at the second step PAPSS2 converts this intermediate to 3′-phosphoadenosine-5′-phosphosulfate. This sulfonate donor is necessary for the constitution of sGAGs of the extracellular matrix.

A comparison of the protein changes between the two different oxygen atmospheres is shown in Fig. 4b. 10 µM of Y-27632 treatment produced changes in 31 proteins, with a similar profile of up- and down-regulations both at normoxia and hypoxia. These 31 proteins are suggested to be Y-27632-induced oxygen-independent responses. Approximately 70% of Y-27632-induced protein fold changes were connected to prevailing oxygen tension.

### qRT-PCR analyses

**Figure 5:**
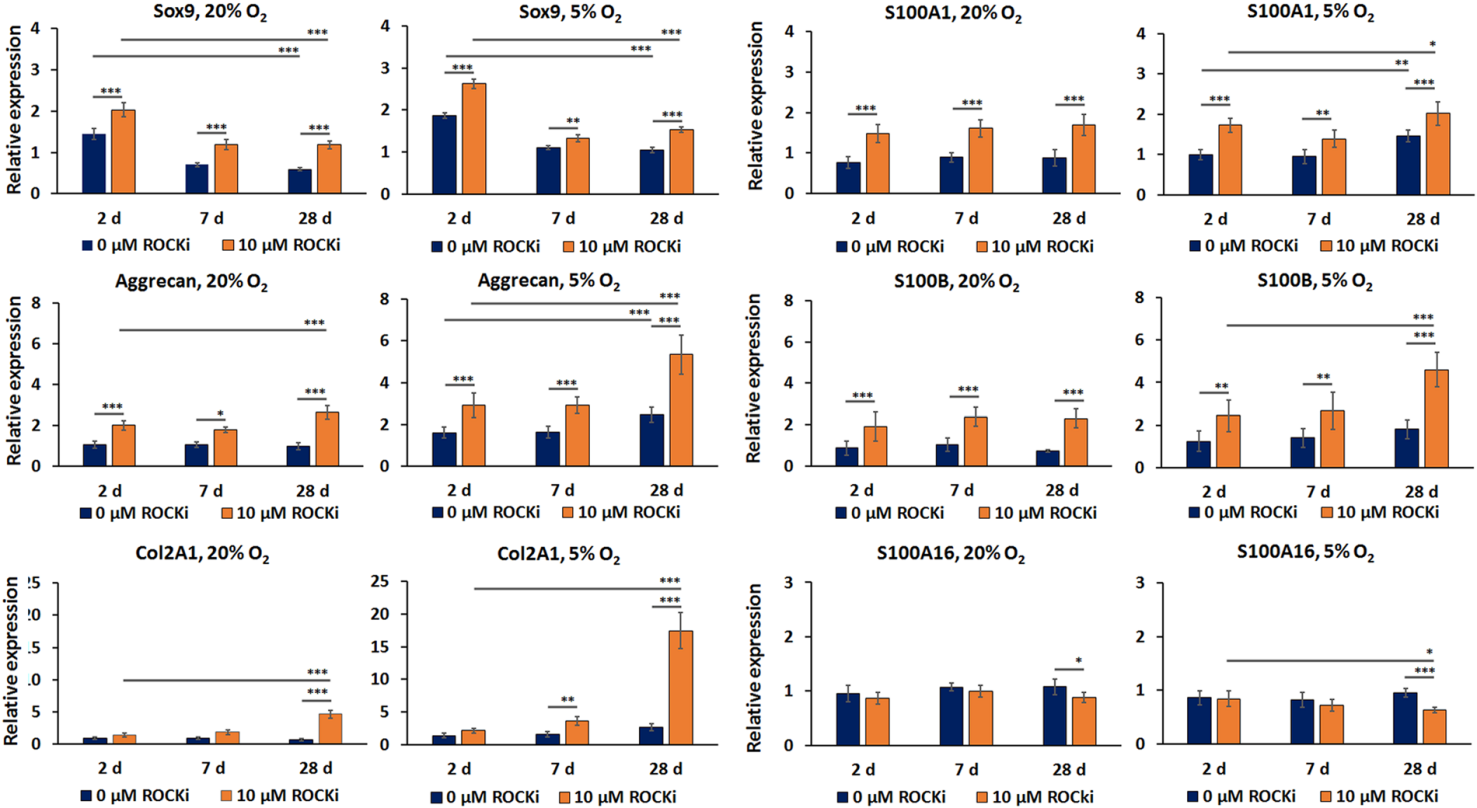
Relative gene expression of SOX9, aggrecan, type II collagen, S100A1, S100A16, S100B at 2, 7 and 28 days of exposure to Y-27632 at normoxia and at hypoxia. The columns describe the mean ± 95% confidence intervals, *n* = 3 with two replicates. (*p ≤ 0.05, **p < 0.01, ***p < 0.001).

Y-27632 treatment increased transcription factor SOX9 gene expression at normoxia and at hypoxia (Fig. 5). SOX9 gene expression was strongest at beginning of the experiments. However, the largest relative differences were obtained at 28 days of prolonged exposure to Y-27632. Treatment with 10 µM Y-27632 also induced ACAN gene expression in both atmospheres (Fig. 5). The highest ACAN gene expression was observed after prolonged treatment at hypoxia for 28 days. Relative expression levels of ACAN in Y-27632-treated cultures at hypoxia were 5.33 ± 0.93 fold (p < 0.001) of those in control cultures. Gene expression of another fibroblast-associated hyaluronan-binding lectican, VCAN, could not be quantified due to low expression levels (data not shown).

COL2A1 expression was significantly increased under both atmospheric conditions after four-week exposure to Y-27632 (Fig. 5). At normoxia, the relative COL2A1 gene expression was 7.07 ± 0.97 fold higher (p < 0.001) in Y-27632-treated cultures than in the controls. Under hypoxic conditions, prolonged kinase inhibitor treatment induced an additional 6.53 ± 1.01 times (p < 0.001) higher gene expression than at normoxia. The combination of hypoxia and Y-27632 synergistically augmented COL2A1 gene expression. Relative expression levels of COL2A1 gene expression at hypoxia to the corresponding controls at normoxia revealed a 26.6 ± 4.14 fold (p < 0.001) increase by 10 µM Y-27632 after 4 weeks in culture.

The proteomic analysis revealed changes in many different S100 protein family members. Therefore, we evaluated whether changes in gene expression of S100-A1, S100-B and S100-A16 also were effected. 10 µM Y-27632 treatment induced a significant increase in S100-A1 gene expression at both oxygen atmospheres at all of the time points measured (Fig. 5). Y-27632 treatment also induced S100-B gene expression both at normoxia and at hypoxia, but with relatively higher changes than with S100-A1 (Fig. 5). Therefore, a four-week exposure of cultures to Y-27632 at hypoxia was the most effective way to increase S100-B gene expression in HCS-2/8 cells. Notably, S100-B gene expression profiles shared a close similarity to ACAN gene expression profiles. In addition, COL2A1 gene expression followed rather well S100B gene expression, albeit with significantly smaller increases. In contrast, S100-A16 gene expression significantly decreased when Y-27632 treatment was continued at hypoxia for 28 days (Fig. 5) with a difference in gene expression of 0.67 ± 0.06 (p < 0.001) between treated cultures and controls.

**Figure 6:**
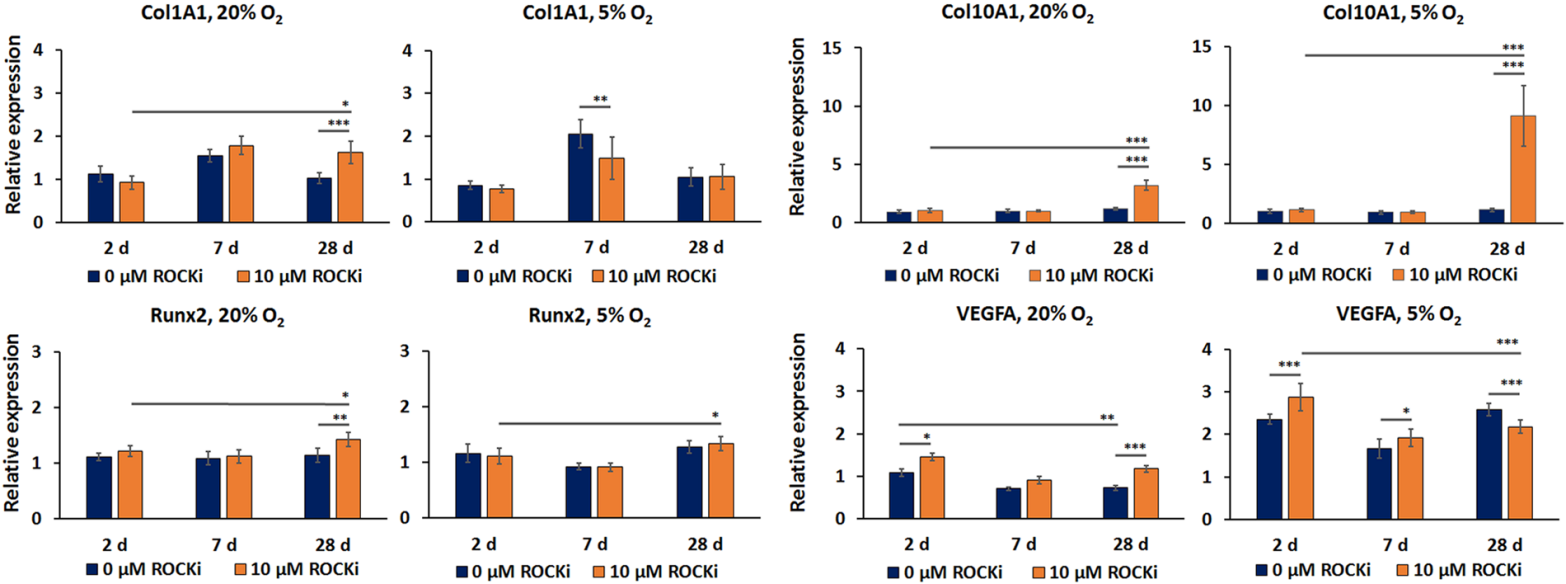
Relative gene expression of type I collagen, type X collagen, Runx2 and VEGFA at 2, 7 and 28 days of exposure to Y-27632 at normoxia and at hypoxia. The columns describe the mean ± 95% confidence intervals, *n* = 3 with two replicates. (*p ≤ 0.05, **p < 0.01, ***p < 0.001).

In addition, Y-27632 treatment induced some changes to the COL1A1 gene expression (Fig. 6). However, the changes in COL1A1 gene expression were nominal in comparison to its effect on COL2A1 expression levels. Taking this into account, we suggest that COL1A1 gene expression had only minor, if any, contribution to extracellular matrix production and composition, or dedifferentiation phenotype. The expression of procollagen α_1_(X) increased significantly during the long-term presence of ROCK-inhibitor, suggesting hypertrophic differentiation of the HCS-2/8 cells (Fig. 6), which is in contrast to previously published data on human chondrocytes^4^. However, the relative ratio of procollagen α_1_(X) to procollagen α_1_(II) remained unchanged in the presence of Y-27632.

The gene expression of Runt-related transcription factor 2 (Runx2) remained stable in all experimental conditions. The highest fold change, 1.26 ± 0.10, was observed between the sample and the control when 10 µM Y-27632 treatment was performed for 28 days at hypoxia. The corresponding induction was not observed at hypoxia. The continuous ROCK-inhibitor exposure induced a slight Runx2 gene expression in the course of time at both oxygen environments. However, the total increase of gene expression were equal or less than 22%. Alone these slight increases are not considered sufficient to induce osteogenic differentiation.

A hypoxic atmosphere has been previously shown to induce VEGFA gene expression in chondrocytes and HCS-2/8 cells^17, 18^. Its gene expression was used as a hypoxia-sensitive control to confirm efficacy of the low oxygen atmosphere used during our experiments. The hypoxic atmosphere induced significantly higher VEGFA gene expression both in control cells and Y-27632 treated cells in compared to the normoxic atmosphere (Fig. 6).

### Type II collagen and sGAG assays

**Figure 7:**
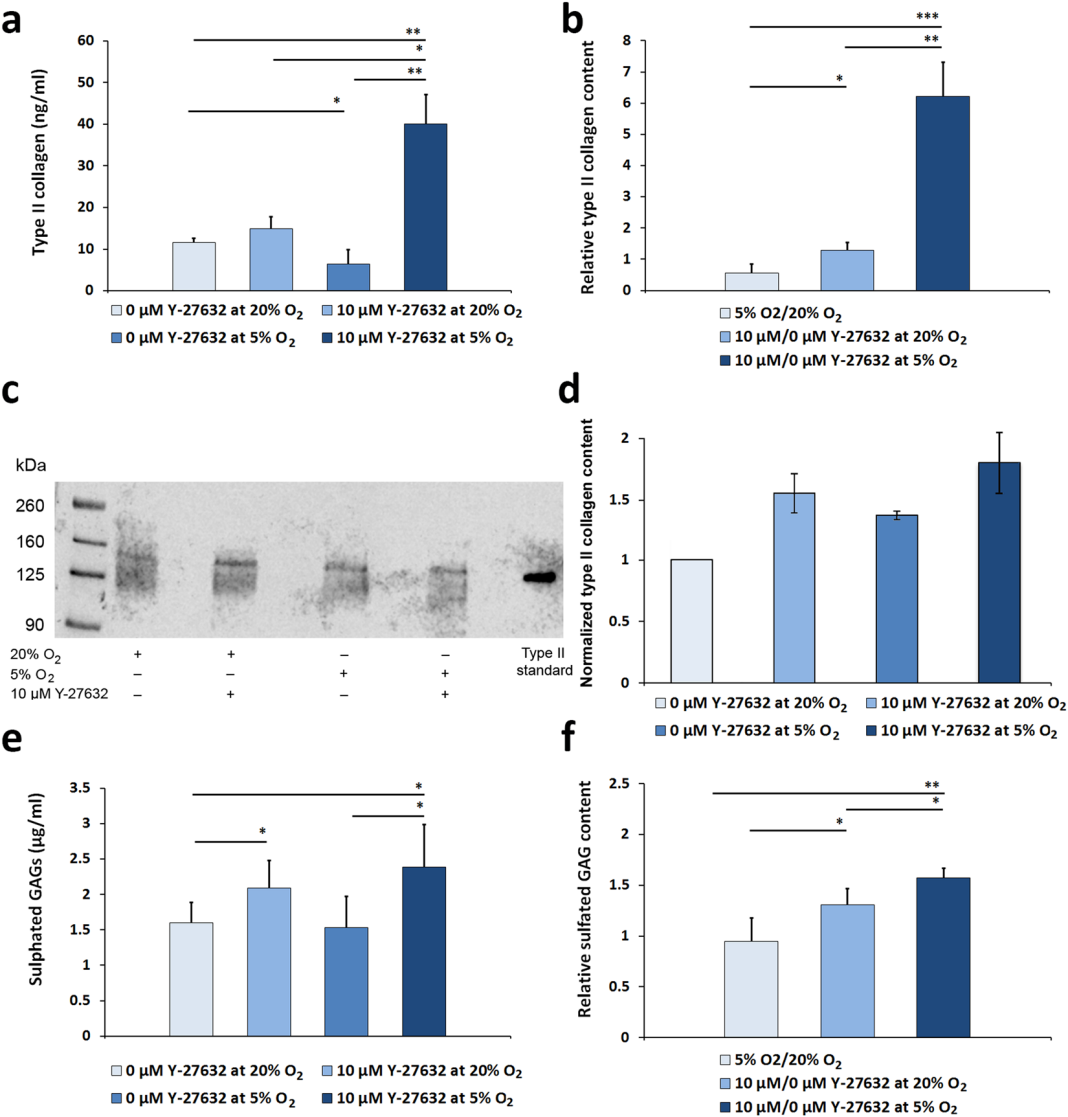
The mean concentration of type II collagen in medium (**a**) and the relative type II collagen expression at different conditions (**b**) are presented in the upper panels. Immunoblotting of type II collagen (**c**) supported the finding that Y-27632 increases its secretion, as shown by normalized intensities of the detected bands (**d**). The mean concentration of sGAGs (**e**) and their relative expression (**f**) are presented below. Four weeks of 10 µM Y-27632 exposure at 5% low oxygen atmosphere produced the highest up-regulation of type II collagen expression and the synthesis of sGAGs by HCS-2/8 cells. (*n* = 3, *p ≤ 0.05, **p < 0.01, ***p < 0.001).

An ELISA assay was performed on the culture medium from the different conditions collected on the day prior to the end of the 28-day experiment. Data from ELISA assays revealed that 10 µM Y-27632 treatment slightly elevated type II collagen secretion at normoxia (Fig. 7a), although this increase did not reach significance. However, Y-27632 treatment at hypoxia significantly increased type II collagen secretion by 6.2 ± 1.1 fold (Fig. 7a and b). Immunoblotting for type II collagen (Fig. 7c) supported the finding that Y-27632 increases its secretion both at normoxic and hypoxic conditions (Fig. 7d).

The DMMB assay revealed that Y-27632 treatment at a 5% oxygen atmosphere increased sGAG secretion by 57% ± 9% compared to untreated control cells (Fig. 7e). Similar kinase inhibitor treatment at normoxia slightly increased (31% ± 16%) the secretion of sGAGs (Fig. 7e and f). Although DMMB assay results should be interpreted with caution due to possible non-specificity caused by the possible presence of other negatively charged macromolecules, such as DNA and RNA, the results support an increased secretion of sGAGs by Y-27632.

## Discussion

Time-lapse imaging was performed to visualize any rapidly induced morphological changes in HCS-2/8 cells in addition to monitoring cell proliferation and cellular migration. This visual monitoring revealed that 10 µM Y-27632 treatment at early time points inhibited the depolymerization of filamentous actin, as evidenced by the formation of long cellular extensions. A scratch wound migration assay revealed a rapid and transient increase in cellular migration, while the effect on the formation of protrusions stayed relatively stable after the first 12 hours. In contrast, inhibition of Rho-kinase by Y-27632 treatment did not influence absolute cell number at normoxia nor hypoxia.

Many different protocols have been investigated for their potential to guide chondrogenic differentiation, such as combinations of growth factors, mechanical loading and three-dimensional culture systems. The main purpose of this study was to investigate a way to enhance the expression of the major cartilage structural components of articular cartilage, aggrecan and type II collagen. To achieve this goal, we tested the ability of 10 µM Y-27632 to enhance extracellular matrix production in HCS-2/8 cells both at normoxic and hypoxic atmospheres.

The transcription factor SOX9 increases the expression of COL2A1 and ACAN, thereby also enhancing chondrogenesis^19, 20^. We observed an increase in SOX9 gene expression in all Y-27632-treated cultures. A long-lasting Y-27632 treatment (28 days) induced a 7-fold increase in the major SOX9 target gene and COL2A1 gene expression at normoxia. However, this up-regulation of COL2A1 only produced a slight increase in type II collagen secretion. At hypoxia, 10 µM Y-27632 produced a synergistic effect on COL2A1 gene expression that was 6.5 times higher than control levels at hypoxia, but was elevated to 27 times higher when compared to controls at normoxia. This gene up-regulation at hypoxia also induced a 6 time increase in type II collagen secretion, detected in the culture medium compared to hypoxic control cultures. Therefore, we suggest that a 5% low oxygen atmosphere can be efficiently used to enhance Y-27632-induced type II collagen expression and secretion by chondrocytes.

Besides type II collagen, aggrecan is another major macromolecule in native cartilage tissue. Therefore, we also focused on determining the gene expression changes of ACAN, the source of most sGAGs in cartilage. Previously, a 5% low oxygen atmosphere was reported to induce ACAN gene expression in bovine chondrocytes^21^. Moreover, Y-27632 has been reported to up-regulate ACAN gene expression in chondrogenically differentiated ATDC5 cells and chondrocytes^3, 5^. In this study, we observed that a 28-day treatment of HCS-2/8 cells with 10 µM Y-27632 at normoxia induced over 2.5 times up-regulation of ACAN gene and a 30.7% ± 16.0% increase in secreted sGAGs. At hypoxia, the effect was substantially increased. 10 µM Y-27632 induced more than 5 times higher ACAN gene expression in comparison to the controls at normoxia and the production of sGAGs increased to 57.3% ± 9.2%. Due to the significant up-regulation of ACAN gene expression and sGAG secretion we suggest that hypoxia significantly enhances Y-27632-induced ACAN expression. A proteomic analysis revealed that the enzymes PAPSS1 and PAPSS2 (3′-phosphoadenosine 5′-phosphosulfate synthase 1 and 2, respectively) vital for GAG sulfation, were also increased.

The proteomes and secretomes of normal healthy human chondrocytes have been previously described^7, 8, 22^ and proteomic changes related to chondrogenesis and osteoarthritis have also been reported^12, 23, 24^. This study demonstrates that long-lasting inhibition of Rho kinase at normoxia and at hypoxia produces changes in proteins typical for a normal chondrocyte phenotype, while chondrogenesis-related proteins remained unaffected. The protein changes observed were not related to hypertrophic differentiation, osteoarthritis, nor inflammation. Noteworthy, proteomic analysis suggested that one of the osteoarthritis markers, cathepsin B (CTSB)^11, 25^, was decreased by Y-27632 treatment.

A number of changes in S100 protein family members were observed. Y-27632-treatment induced elevations in S100-A1 and S100-B protein levels at both oxygen atmospheres. These increases could be expected since S100-A1 and S100-B genes are targets for SOX9 transcription factor and its coactivators SOX5 and SOX6^26^. Importantly, overexpression of S100-A1 and S100-B genes has been related to a suppression of the hypertrophic differentiation of chondrocytes and mineralization process^26^. The inhibition of the terminal differentiation of chondrocytes is favorable since this will maintain cartilage-specific extracellular matrix production and functional structure. However, the overexpression of S100-A1 and S100-B cannot induce the early stage of chondrocyte differentiation thereby, additional inducers are required for chondrogenic differentiation of mesenchymal stem cells. Our long-term (28 days) S100-A1 and S100-B findings support the theory that Y-27632 may strengthen a chondrocyte phenotype.

Protein S100-A13 was another S100 protein influenced by Y-27632 at both atmospheres. S100-A13 participates in stress-induced fibroblast growth factor 1 signaling^27^. However, no cartilage-specific function of S100-A13 has been described to date. Still, roughly a two-fold increase was observed and suggests that further investigations are necessary to reveal potential chondrocyte-specific S100-A13 function.

Y-27632 treatment elevated S100-A4 levels at normoxia, but not at hypoxia. This protein binds to receptor for advanced glycation end products (RAGE), and mediates the induction of MMP13 in articular chondrocytes^28^. Since osteoarthritis increases S100-A4 expression and MMP13 expression, we suggest that the lack of Y-27632-induced S100-A4 levels under hypoxic conditions may beneficially decrease the risk of MMP13 -mediated degradation of extracellular matrix.

Y-27632 was found to decrease S100-A6 protein levels at hypoxia, but not at normoxia, which suggests an oxygen-dependent regulation. BMP4-stimulated chondrogenic induction in the ATDC5 cell line was previously reported to down-regulate S100-A6 gene expression^29^. The link between chondrogenic induction and down-regulation of S100-A6 protein could have a connection to the increased extracellular matrix production we observed in HCS-2/8 cells. The calcium sensitive S100-A6 mediates additional responses, such as cellular proliferation, apoptosis, cytoskeletal dynamics and tumorigenesis^30, 31^.

Proteins S100-A1, S100-A2, S100-A6 and S100-B are known to activate serine/threonine protein phosphatase 5 tetratricopeptide repeat domain (PP5-TRP)^30^. Interaction of S100 proteins with PP5-TRP participate to fully activate the enzyme. PP5 has been indicated in processes involved with several phosphorylation cascades, proliferation, migration, differentiation, apoptosis, DNA damage repair and electrolyte balance^30, 32^. Consequently, it may have biological targets in chondrocytes still to be discovered.

Amino acid transporters SLC7A2 (LAT1, CD98LC) and SLC3A2 (4F2hc, CD98HC)^33, 34^ were both remarkably decreased at normoxia and hypoxia. Interestingly, previous studies have revealed a correlation between LAT1 and prognosis of different cancers. There have been speculations on the possibility to use amino acid transporters as therapeutic targets to inhibit tumor progression^35–37^. Recent studies have addressed a key role of LAT1 for growth limiting activities, which are mediated by the LAT1/4F2hc transporter. Therefore, LAT1 was classified as a potent inhibition target for novel cancer treatment development^38, 39^. The Rho kinase inhibitor Y-27632 induced a down-regulation of LAT1/4F2hc heterodimer components, previously unreported, and this finding might offer new possibilities for Rho-kinase inhibition in cancer research. In this context, we suggest that a down-regulation of LAT1/4F2hc transporter most probably restricted the transport of certain essential amino acids. However, due to an increase in extracellular matrix production at hypoxia, it is evident that the LAT1/4F2hc transporter was not the only mechanism for essential amino acid uptake by HCS-2/8 cells. In future studies, it is important to investigate how LAT1/4F2hc down-regulation affects the maintenance of chondrocyte phenotype and whether it has a role in defining the status of chondrocyte differentiation.

There are many possible mechanisms for the observed findings. Hypoxia and ROCK-inhibitor treatment led to a high number of changes in gene and protein expressions. Many of the hypoxia-related effects most likely involve activation of HIF pathways, which involve a high number of downstream-target genes^40^. S100 proteins make up an interesting group of Ca^2+^-binding proteins, which involve the regulation of chondrocyte function. The functional studies of S100 proteins in chondrocytes have been mainly to investigate their extracellular roles^41^. In chondrocytes, S100-A1 and S100-B are transcriptional targets of SOX trio (SOX5, SOX6 and SOX9), and their expression has been suggested to inhibit terminal differentiation of chondrocytes^26^. Our results are in line with a previous study showing that S100-A1 gene expression closely correlates with that of SOX9^42^.

Possible mechanism for S100 protein actions may involve TGF-β/SMAD, cyclic AMP (cAMP)/protein kinase A (PKA)/cAMP response element binding protein (CREB), phosphoinositide-3 kinase (PI3K)/Akt, Ca^2+^ homeostasis, calcineurin/NFAT and RAGE related pathways, as reviewed recently^43^. TGF-β is known to stimulate the early events in chondrogenesis and inhibit terminal hypertrophic chondrocyte differentiation, and S100-A1 and S100-B may modulate this signaling via the interactions of PP5-TRP^30^. cAMP induces transcription through PKA and phosphorylation of CREB^44^. Further, PKA is known to phosphorylate SOX9 and increase its DNA binding and transcriptional activities^45^, and CREB can also enhance SOX9 activity^46^. PI3K/Akt pathway is known to potentially increase chondrogenic genes and to regulate chondrocyte terminal differentiation^47^. S100-A1 can regulate cellular Ca^2+^ homeostasis through, e.g., ryanodine receptor^48^. Transient receptor potential family transporters are also important Ca^2+^ balance regulators. In chondrocytes, vanilloid subfamily 4 (TRPV4) in particular, participates in cartilage maintenance by regulating SOX9 expression. S100-A1 can regulate TRPV4 indirectly by activating PKA^49^, but direct activation can also be possible via calmodulin binding site.

In conclusion, the Rho-kinase inhibitor Y-27632 efficiently enhanced extracellular matrix production in cultured chondrocytic cells under hypoxic conditions. This treatment may be useful in applications that require long culture periods for the expansion of chondrocyte cell number and/or to generate tissue structure for cartilage repair. This study supports the previous finding that S100-A1 and S100-B proteins correlate with the differentiation status of chondrocytes that could be used as putative markers for chondrogenic extracellular matrix production^50^. Noteworthy, S100-B protein gene expression profile had close similarity with ACAN gene expression profiles in all conditions tested. In addition to up-regulation of S100-A1 and S100-B proteins, Rho-kinase inhibition produced other changes in S100 protein family members at both oxygen atmospheres. The exact function and possible chondrocyte-phenotype supporting mechanisms of these proteins are still unknown and warrant further investigation. Although the HCS-2/8 cell line shares features of normal chondrocytes, differences have also been detected^14^. Therefore, it will be important to validate the findings with primary human chondrocytes, preferably in three-dimensional culture systems, since monolayer cultures maintained for 4 weeks also dedifferentiate and change their gene expression patterns, reducing their value as reference for normal chondrocyte phenotype.

## Methods

### Human chondrosarcoma 2/8 cell culture

HCS-2/8 cells^13^ were cultured in high glucose (4.5 g/l) DMEM (Sigma-Aldrich), supplemented with 10% FBS (Lonza), 100 U/ml of penicillin (Lonza), 100 µg/ml of streptomycin (Lonza), 2 mM L-glutamine (Lonza) and 50 µg/ml 2-phospho-L-ascorbic acid trisodium salt (Sigma-Aldrich). Cells were cultured at 37 °C either in a humidified incubator at normoxia or at a 5% oxygen atmosphere in a Galaxy R incubator (RS Biotech Laboratory Equipment Ltd).

Slowly proliferating HCS-2/8 cells were seeded at a density of 200,000 cells per/well in 6-well-plates (Greiner Bio-One GmbH) for time-lapse imaging assays. The proliferation and the morphology assays were started one hour after plating cells. The scratch wound assays were performed on 90% confluent monolayer cultures. A concentration of 10 µM Y-27632 (Sigma) was used in culture. The medium was not changed during the time-lapse experiments and imaging experiments were performed both at normoxic and 5% oxygen atmospheres. The cell monitoring was repeated three times with at least two replicate grids, and the scratch wound assay was repeated three times with the six replicate grids at both oxygen atmospheres.

HCS-2/8 cells were also seeded in 6-well-plates for quantitative reverse transcription-polymerase chain reaction (qRT-PCR) gene expression analyses. 900,000 cells were seeded per/well for the two-day experiment, 400,000 cells for the seven-day experiment and 200,000 cells for the 28-day experiment. These cell densities were used to reach 90% confluency prior to sample collection at 2, 7, and 28 days. The cells of the 28 days experiment were passaged once after the two weeks culturing period. Y-27632 at 10 µM was prepared in fresh culture medium while controls were cultured without Y-27632. During the experiments, the culture medium was removed every third day and fresh culture media with, or without Y-27632 was added. The experiments were performed at 20% and 5% oxygen atmospheres. For hypoxia experiments, fresh culture medium was equilibrated to a 5% oxygen atmosphere for 24 hours before its addition to cell cultures. Y-27632 was added to culture medium immediately prior to its use. The experiments were repeated three times in duplicate.

For the analysis of the proteome, 275,000 cells were seeded in 9.5 cm culture dishes and treated with Y-27632 during four weeks. The experiments were repeated three times at both oxygen atmospheres.

### Cell monitoring using time-lapse imaging

Time-lapse imaging was used as a method to determine 10 µM Y-27632-induced effects on cellular proliferation, morphology and migration of HCS-2/8 cells. Analyses were performed using the Cell-IQ microscope system (Cell-IQ Model v.2., CM Technologies). 37 °C atmospheres at normoxia (5% CO_2_, 19% O_2_ and 76% N_2_) or at 5% oxygen (5% CO_2_, 5% O_2_ and 90% N_2_) were maintained using premixed gases (Aga) throughout the entirety of the experiments. For proliferation and morphology assays, average cell densities of 85 cells per each monitored grid (984 µm × 732 µm) were seeded at the beginning of each experiment. An interval of one hour was used between images and cells were monitored over 72 hours by phase-contrast microscopy. Experiments were repeated three times using at least two replicate grids per experiment. Total cell numbers and the length of cellular extensions were estimated using shape recognition algorithms as part of the Cell-IQ analyser 4 Pro-write (AN4.2.1 HW, CM Technologies) software. Any random errors in the automatic cell recognition were corrected manually.

This time-lapse imaging method was also applied to a scratch wound assay. Scratch wounds were performed on cell monolayers using a narrow pipette tip. Y-27632 was added at 10 µM immediately before the assay. An interval of one hour was used between images and cells were monitored over 60 hours by phase-contrast microscopy. Due to the slow migration of cells, data was analyzed manually at the twelve-hour interval.

### Proteomic analyses and data interpretation

Proteins were extracted and trypsin-digested closely following published procedures^51^, and the resulting peptides were cleaned using a C18 STAGE-tip^52^. After cleaning, the peptide concentration of each sample was determined using a micro BCA kit (Thermo Fisher Scientific). Approximately 600 ng of each sample was loaded onto a HSS T3 C18 analytical column (75 μm i.d. × 200 mm, 1.8 μm particles; Waters, Milford, MA, USA), and separated using a linear 108 min gradient of 5–41% solvent B (3:1 acetonitrile/2-propanol) balanced with 0.1% aqueous formic acid (solvent A) at a flow rate of 295 nl min^−1^. The eluate was passed to a nano-ESI equipped SynaptTM G2-Si HDMS mass spectrometer (Waters) operating in resolution mode. All data were collected using ion-mobility MS^E^ with a scan-time of 0.5 sec and mass-corrected using Glu-fibrinopeptide B and Leucine Enkephalin as reference peptides. Data was analyzed using ProgenesisQI (Nonlinear Dynamics), and the peptides were identified using the built-in MS^E^ search option, employing a false discovery rate of <1% with a mass error cut-off of 10 ppm. Two missed cleavages where allowed, carbamidomethylated cysteines used as fixed modification, and both oxidization of methionine, and deamidation of asparagine and glutamine as variable modifications. Protein abundances were calculated using the Hi-N method of the software (*n* = 3). The functional roles of the quantitated proteins were interpreted with Ingenuity Pathway Analysis (IPA^®^, Ingenuity Systems Inc., Qiagen Hilden, Germany). Proteomics data are deposited in PRIDE archive (project accession: PXD005551).

### qRT-PCR analyses

Total RNA extraction and sample preparation steps prior to qRT-PCR analyses were performed as previously described^15^. qRT-PCR reactions were performed with CFX Connect™ Real-Time PCR Detection System (Bio-Rad Laboratories). 4 ng of cDNA was used in each qRT-PCR reaction. The specific primer sequences are presented in Supplementary Table 1 ^53–63^. Primers were purchased from Oligomer (Oligomer Oy) and from Invitrogen (Thermo Fisher Scientific). qRT-PCR was performed using Maxima SYBR Green/ROX qPCR Master Mix (Thermo Fisher Scientific) according to the manufacturer’s instructions. qRT-PCR reaction conditions were based on previously used parameters^15^, or optimized for the new primer pairs accordingly. The amplification efficiency for each primer pair was determined from the slopes of standard curves using gradient concentrations of cDNA. All primer pairs had efficiencies between 90–110%. RPLP0 was used as the housekeeping gene for normalization. The specificities of the PCR products were determined by post-PCR melting curve analysis and subsequently visualized on a 2.5% agarose gel.

### Determination of the type II collagen by ELISA analysis

The quantification of the type II collagen from culture medium was performed using the commercial ELISA Type II Collagen Detection Kit (#6018, Chondrex). The analysis was performed according to the manufacturer’s instructions. The absorbances were determined at 490 nm using a Vmax kinetic microplate reader (Molecular Devices) following a one-hour-incubation in *o*-phenylenediamine solution.

### Immunoblotting of type II collagen

Samples prepared with Laemmli buffer were heated 2 h at 50 °C and resolved by SDS-PAGE using Mini-PROTEAN^®^ TGX™ Precast Protein 4–15% gradients gels (Bio-Rad). Chicken type II collagen (Chondrex) was used as a reference to validate its immunodetection from media taken from tissue culture supernatants. Proteins were transferred to polyvinyl (PVDF) membranes using the Trans-Blot Turbo western blotting transfer system (Bio-Rad). PVDF membranes were stained with Revert Total Protein (Licor) and blocked for 1 h in 1X Tris-buffered saline (TBS) with 5% non-fat milk (Bio-Rad) at room temperature. PVDF membranes were incubated overnight at 4 °C with a mouse monoclonal anti-type II collagen antibody (10 µg/ml, #7005, Chondrex) according to the manufacturer’s indications. Three times washes were done for 10 min each in 1X TBS with 0.1% Tween-20 previous detection using the corresponding peroxidase-conjugated anti-mouse antibody for 1 h at room temperature. Type II collagen was detected with the ECL supra bright kit (Agrisera). Chemiluminescent signal on PVDF membranes was detected using the Odyssey Fc Imaging system (Licor), and the intensities of the bands after removal of background intensities were quantified using ImageStudioLite software (Licor) and normalized to the total amount of protein in each lane.

### Dimethylmethylene Blue assay (DMMB) for sulfated glycosaminoglycans (sGAGs)

The quantity of sGAGs in culture medium was evaluated using the DMMB assay that was slightly modified from the originally described protocol^64^. Before the analyses, medium samples were centrifuged to eliminate any detached cells from the medium. Culture medium samples (75 µl) were rapidly mixed with 250 µl DMMB reagent in a 96-well-plate and the absorbance at 535 nm were instantly measured using a Vmax kinetic microplate reader. Shark chondroitin sulfate (Sigma-Aldrich) prepared in fresh culture media was used as a standard. Linearity was tested in a range from 0 to 5.77 µg/ml, in which R^2^-values ≥ 0.97 were achieved. The background noise of the medium matrix was measured and the detection limit of the analyte (LOD) was determined on the basis of the limit of blank (LOB)^65^. Five independent blank samples and 15 low concentration samples were used to determine LOB and LOD. The arbitrary limit of quantification (LOQ) was not stated for this semiquantitave assay. However, all sGAG concentrations compared were at least three times higher than the limit of detection (0.35 µg/ml).

### Statistical analysis

Statistical analysis for time-lapse imaging data was performed with one-way ANOVA test (SPSS, v.21, IBM). The statistical significance of the mass spectrometry-based protein quantifications were determined from the normalized arcsinh transformed data with one-way ANOVA tests using the ProgenesisQI software (Nonlinear Dynamics). One-way ANOVA with Fisher’s Least Significant Difference (LSD) *post-hoc* test was used for qRT-PCR data analysis. A Student’s *t*-test was used for the paired data analysis of the results obtained from ELISA and DMMB methods. The *p*-values equal or less than 0.05 were considered significant.

## Electronic supplementary material

Supplementary Inoformation

## References

[1] Villar-Suárez V (2004). Differential behavior between isolated and aggregated rabbit auricular chondrocytes on plastic surfaces. J Biomed Biotechnol.

[2] Tay LX, Lim CK, Mansor A, Kamarul T (2014). Differential protein expression between chondrogenic differentiated MSCs, undifferentiated MSCs and adult chondrocytes derived from Oryctolagus cuniculus *in vitro*. Int J Med Sci.

[3] Woods A, Beier F (2006). RhoA/ROCK signaling regulates chondrogenesis in a context-dependent manner. J Biol Chem.

[4] Matsumoto E, Furumatsu T, Kanazawa T, Tamura M, Ozaki T (2012). ROCK inhibitor prevents the dedifferentiation of human articular chondrocytes. Biochem Biophys Res Commun.

[5] Furumatsu T, Matsumoto-Ogawa E, Tanaka T, Lu Z, Ozaki T (2014). ROCK inhibition enhances aggrecan deposition and suppresses matrix metalloproteinase-3 production in human articular chondrocytes. Connect Tissue Res.

[6] Grassel S, Ahmed N (2007). Influence of cellular microenvironment and paracrine signals on chondrogenic differentiation. Front Biosci.

[7] Ruiz-Romero C, López-Armada MJ, Blanco FJ (2005). Proteomic characterization of human normal articular chondrocytes: a novel tool for the study of osteoarthritis and other rheumatic diseases. Proteomics.

[8] Lambrecht S (2010). Proteome characterization of human articular chondrocytes leads to novel insights in the function of small heat-shock proteins in chondrocyte homeostasis. Osteoarthritis Cartilage.

[9] Lee SJ (2004). Identification of proteins differentially expressed during chondrogenesis of mesenchymal cells. FEBS Lett.

[10] de la Fuente A (2012). Proteome analysis during chondrocyte differentiation in a new chondrogenesis model using human umbilical cord stroma mesenchymal stem cells. Mol Cell Proteomics.

[11] Tsolis KC (2015). Comparative proteomic analysis of hypertrophic chondrocytes in osteoarthritis. Clin Proteomics.

[12] Ruiz-Romero C (2008). Proteomic analysis of human osteoarthritic chondrocytes reveals protein changes in stress and glycolysis. Proteomics.

[13] Takigawa M (1989). Establishment of a clonal human chondrosarcoma cell line with cartilage phenotypes. Cancer Res.

[14] Saas J, Lindauer K, Bau B, Takigawa M, Aigner T (2004). Molecular phenotyping of HCS-2/8 cells as an *in vitro* model of human chondrocytes. Osteoarthritis Cartilage.

[15] Piltti J, Varjosalo M, Qu C, Häyrinen J, Lammi MJ (2015). Rho-kinase inhibitor Y-27632 increases cellular proliferation and migration in human foreskin fibroblast cells. Proteomics.

[16] Venkatachalam KV (2003). Human 3′-phosphoadenosine 5′-phosphosulfate (PAPS) synthase: biochemistry, molecular biology and genetic deficiency. IUBMB Life.

[17] Lin C (2004). Hypoxia induces HIF-1alpha and VEGF expression in chondrosarcoma cells and chondrocytes. J Orthop Res.

[18] Kondo S (2006). Hypoxic regulation of stability of connective tissue growth factor/CCN2 mRNA by 3′-untranslated region interacting with a cellular protein in human chondrosarcoma cells. Oncogene.

[19] Bell DM (1997). SOX9 directly regulates the type-II collagen gene. Nat Genet.

[20] Sekiya I (2000). SOX9 enhances aggrecan gene promoter/enhancer activity and is up-regulated by retinoic acid in a cartilage-derived cell line, TC6. J Biol Chem.

[21] Qu CJ, Pöytäkangas T, Jauhiainen M, Auriola S, Lammi MJ (2009). Glucosamine sulphate does not increase extracellular matrix production at low oxygen tension. Cell Tissue Res.

[22] Polacek M, Bruun JA, Johansen O, Martinez I (2010). Differences in the secretome of cartilage explants and cultured chondrocytes unveiled by SILAC technology. J Orthop Res.

[23] Ji YH (2010). Quantitative proteomics analysis of chondrogenic differentiation of C3H10T1/2 mesenchymal stem cells by iTRAQ labeling coupled with on-line two-dimensional LC/MS/MS. Mol Cell Proteomics.

[24] Lambrecht S, Verbruggen G, Verdonk PC, Elewaut D, Deforce D (2008). Differential proteome analysis of normal and osteoarthritic chondrocytes reveals distortion of vimentin network in osteoarthritis. Osteoarthritis Cartilage.

[25] Lai WF, Chang CH, Tang Y, Bronson R, Tung CH (2004). Early diagnosis of osteoarthritis using cathepsin B sensitive near-infrared fluorescent probes. Osteoarthritis Cartilage.

[26] Saito T, Ikeda T, Nakamura K, Chung UI, Kawaguchi H (2007). S100A1 and S100B, transcriptional targets of SOX trio, inhibit terminal differentiation of chondrocytes. EMBO Rep.

[27] Sivaraja V (2006). Copper binding affinity of S100A13, a key component of the FGF-1 nonclassical copper-dependent release complex. Biophys J.

[28] Sparvero LJ (2009). RAGE (Receptor for Advanced Glycation Endproducts), RAGE ligands, and their role in cancer and inflammation. J Transl Med.

[29] Wahl M (2004). Transcriptome analysis of early chondrogenesis in ATDC5 cells induced by bone morphogenetic protein 4. Genomics.

[30] Yamaguchi F (2012). S100 proteins modulate protein phosphatase 5 function: a link between CA2+ signal transduction and protein dephosphorylation. J Biol Chem.

[31] Donato R (2013). Functions of S100 proteins. Curr Mol Med.

[32] Golden T, Swingle M, Honkanen RE (2008). The role of serine/threonine protein phosphatase type 5 (PP5) in the regulation of stress-induced signaling networks and cancer. Cancer Metastasis Rev.

[33] Yanagida O (2001). Human L-type amino acid transporter 1 (LAT1): characterization of function and expression in tumor cell lines. Biochim Biophys Acta.

[34] Fotiadis D, Kanai Y, Palacín M (2013). The SLC3 and SLC7 families of amino acid transporters. Mol Aspects Med.

[35] Furuya M, Horiguchi J, Nakajima H, Kanai Y, Oyama T (2012). Correlation of L-type amino acid transporter 1 and CD98 expression with triple negative breast cancer prognosis. Cancer Sci.

[36] Kaira K (2012). Prognostic significance of L-type amino-acid transporter 1 expression in surgically resected pancreatic cancer. Br J Cancer.

[37] Betsunoh H (2013). Increased expression of system large amino acid transporter (LAT)-1 mRNA is associated with invasive potential and unfavorable prognosis of human clear cell renal cell carcinoma. BMC Cancer.

[38] Cormerais Y (2016). Genetic disruption of the multifunctional CD98/LAT1 complex demonstrates the key role of essential amino acid transport in the control of mTORC1 and tumor growth. Cancer Res.

[39] Marshall AD (2016). LAT1 is a putative therapeutic target in endometrioid endometrial carcinoma. Int J Cancer.

[40] Benita Y (2009). An integrative genomics approach identifies Hypoxia Inducible Factor-1 (HIF-1)-target genes that form the core response to hypoxia. Nucleic Acids Res.

[41] Yammani RR (2012). S100 proteins in cartilage: role in arthritis. Biochim Biophys Acta.

[42] Tew SR, Cleqq PD, Brew CJ, Redmond CM, Hardingham TE (2007). SOX9 transduction of a human chondrocytic cell line identifies novel genes regulated in primary human chondrocytes and in osteoarthritis. Arthritis Res Ther.

[43] Diaz-Romero J, Nesic D (2017). S100A1 and S100B: Calcium sensors at the cross-roads of multiple chondrogenic pathways. J Cell Physiol.

[44] Zhao L, Li G, Zhou GC (2009). SOX9 directly binds CREB as a novel synergism with the PKA pathway in BMP-2-induced osteochondrogenic differentiation. J Bone Miner Res.

[45] Furumatsu T, Tsuda M, Taniguchi N, Tajima Y, Asahara H (2005). Smad3 induces chondrogenesis through the activation of SOX9 via CREB-binding protein/p300 recruitment. J Biol Chem.

[46] Mayr B, Montminy M (2001). Transcriptional regulation by the phosphorylation-dependent factor CREB. Nat Rev Mol Cell Biol.

[47] Kita K, Kimura T, Nakamura N, Yoshikawa H, Nakano T (2008). PI3K/Akt signaling as a key regulatory pathway for chondrocyte terminal differentiation. Genes Cells.

[48] Wright NT, Cannon BR, Zimmer DB, Weber DJ (2009). S100A1: Structure, Function, and Therapeutic Potential. Curr Chem Biol.

[49] Fan HC, Zhang X, McNaughton PA (2009). Activation of the TRPV4 ion channel is enhanced by phosphorylation. J Biol Chem.

[50] Diaz-Romero J (2014). S100A1 and S100B expression patterns identify differentiation status of human articular chondrocytes. J Cell Physiol.

[51] Masuda T, Tomita M, Ishihama Y (2008). Phase transfer surfactant-aided trypsin digestion for membrane proteome analysis. J Proteome Res..

[52] Rappsilber J, Ishihama Y, Mann M (2003). Stop and go extraction tips for matrix- assisted laser desorption/ionization, nanoelectrospray, and LC/MS sample pretreatment in proteomics. Anal Chem.

[53] Qu C (2013). Chondrogenic differentiation of human pluripotent stem cells in chondrocyte co-culture. Int J Biochem Cell Biol.

[54] Martin I (2001). Quantitative analysis of gene expression in human articular cartilage from normal and osteoarthritic joints. Osteoarthritis Cartilage.

[55] Caron MM (2013). Hypertrophic differentiation during chondrogenic differentiation of progenitor cells is stimulated by BMP-2 but suppressed by BMP-7. Osteoarthritis Cartilage.

[56] Jokela TA (2011). Cellular content of UDP-N-acetylhexosamines controls hyaluronan synthase 2 expression and correlates with O-linked N-acetylglucosamine modification of transcription factors YY1 and SP1. J Biol Chem.

[57] Das K (2009). Positive association between nuclear Runx2 and oestrogen-progesterone receptor gene expression characterises a biological subtype of breast cancer. Eur J Cancer.

[58] Eisenbacher JL (2014). S100A4 and uric acid promote mesenchymal stromal cell induction of IL-10+/IDO+ lymphocytes. J Immunol.

[59] Tanaka M (2015). Co-expression of S100A14 and S100A16 correlates with a poor prognosis in human breast cancer and promotes cancer cell invasion. BMC Cancer.

[60] Leclerc E, Heizmann CW, Vetter SW (2009). RAGE and S100 protein transcription levels are highly variable in human melanoma tumors and cells. Gen Physiol Biophys.

[61] Li Y (2004). Transduction of passaged human articular chondrocytes with adenoviral, retroviral, and lentiviral vectors and the effects of enhanced expression of SOX9. Tissue Eng.

[62] Löfstedt T (2004). Induction of ID2 expression by hypoxia-inducible factor-1: a role in dedifferentiation of hypoxic neuroblastoma cells. J Biol Chem.

[63] Pattyn F, Speleman F, De Paepe A, Vandesompele J (2003). RTPrimerDB: the real-time PCR primer and probe database. Nucleic Acids Res.

[64] Farndale RW, Buttle DJ, Barrett AJ (1986). Improved quantitation and discrimination of sulphated glycosaminoglycans by use of dimethylmethylene blue. Biochim Biophys Acta.

[65] Armbruster DA, Pry T (2008). Limit of blank, limit of detection and limit of quantitation. Clin Biochem Rev.

