# Supplementary material for: Rho-kinase inhibitor Y-27632 and hypoxia synergistically enhance chondrocytic phenotype and modify S100 protein profiles in human chondrosarcoma cells
Source: Sci Rep. 2017 Jun 16;7:3708. doi: 10.1038/s41598-017-03958-5 (PMC5473921; doi:10.1038/s41598-017-03958-5)
Supplement: Supplementary file 1 — Supplementary Inoformation [file 41598_2017_3958_MOESM1_ESM.pdf]

Rho-kinase inhibitor Y-27632 and hypoxia synergistically enhance chondrocytic phenotype and modify S100 protein profiles in human chondrosarcoma cells

Juha Piltti[1], Joakim Bygdell[2], Cecilia Fernández-Echevarría[1], Daniel Marcellino[1], & Mikko J. Lammi[1,3]

[1]Department of Integrative Medical Biology, Umeå University, Linnaeus väg 9, 90187 Umeå, Sweden. [2]Department of Chemistry, Umeå University, Linnaeus väg 10, 90187 Umeå, Sweden. [3]School of Public Health, Health Science Center of Xi'an Jiaotong University, Key Laboratory of Trace Elements and Endemic Diseases, National Health and Family Planning Commission, Xi'an, China.

Supplementary table 1. The list of the RT-PCR primer sequences, product sizes (bp) and the references.

Gene	Primer pairs	Product size (bp)	Reference
ACAN	F:5' -CAC TGT TAC CGC CAC TTC CC-3'	303	[53]
	R:5' -AAC ATC ATT CCA CTC GCC CT-3'		
COL1A1	F:5' -CAG CCG CTT CAC CTA CAG C -3'	70	[54]
	R:5' -TTT TGT ATT CAA TCA CTG TCT TGC C -3'		
COL2A1	F:5' -AAG GTC ATG CTG GTC TTG CT-3'	79	[54]
	R:5' -GAC CCT GTT CAC CTT TTC CA-3'		
*COL10A1	F:5' -ATG ATG AAT ACA CCA AAG GCT ACC T -3'	82	[55]
	R:5' -AGC CAC ACC TGG TCA TTT TCT G -3'		
RPLP0	F:5' -AGA TGC AGC AGA TCC GCA T-3'	319	[56]
	R:5' -GTG GTG ATA CCT AAA GCC TG-3'		
RUNX2	F:5' -AGC TTC TGT CTG TGC CTT CTG G -3'	133	[57]
	R:5' - GGA GTG GAC GAG GCA AGA GTT T -3'		
S100A1	F:5' -GCT CTG AGC TGG AGA CGG CG -3'	250	[58]
	R:5' - GCC ACT GTG AGA GCA GCC ACA -3'		
S100A16	F:5' -CAG GGA GAT GTC AGA CTG CTA CAC -3'	358	[59]
	R:5' -CAT CAG GCC AGT GCC TGG AA -3'		
S100B	F:5' -CCG AAC TGA AGG AGC TCA TC -3'	174	[60]
	R:5' -AGA ACT CGT GGC AGG CAG TA -3'		
SOX9	F:5' -GAC TTC CGC GAC GTGGAC-3'	99	[61]
	R:5' -GTT GGG CGG CAG GTA CTG-3'		
VEGFA	F:5' -AGG AGG AGG GCA GAA TCA TCA -3'	76	[62]
	R:5' -CTC GAT TGG ATG GCA GTA GCT -3'		
VCAN	F:5' -CAA GCATCC TGT CTC ACG AA -3'	103	RTprimerDB
	R:5' -CAA CGG AAG TCA TGC TCA AA -3'		ID:271**

*Reverse primer of reference [55] corrected to match human type X collagen; **RT Primer Database [63]

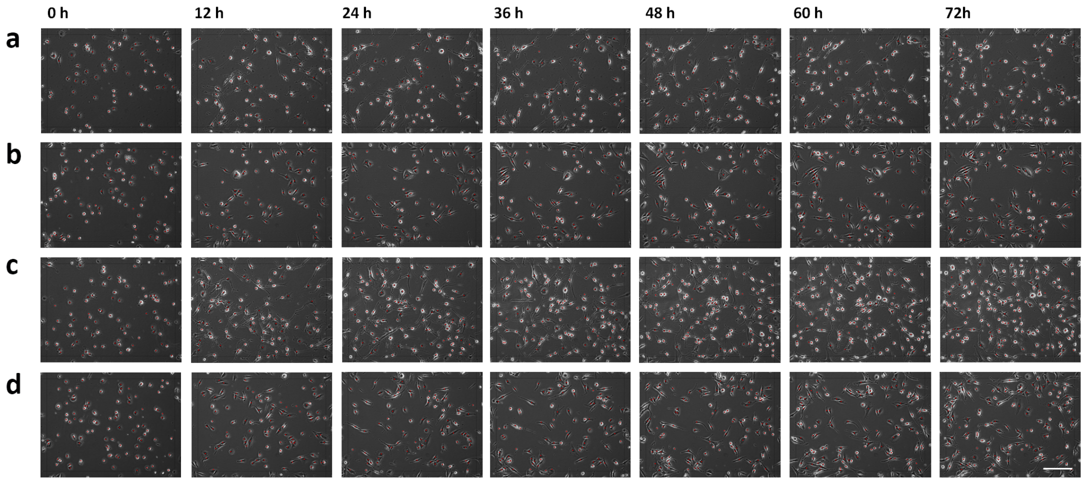

Supplementary Figure 1. Time-lapse images of the HCS-2/8 cells after 0-72 h culture at normoxia in the absence (**a**) and presence (**b**) of 10 μM Y-27632, and at hypoxia (5% oxygen) in the absence (**c**) and presence (**d**) of 10 μM Y-27632.

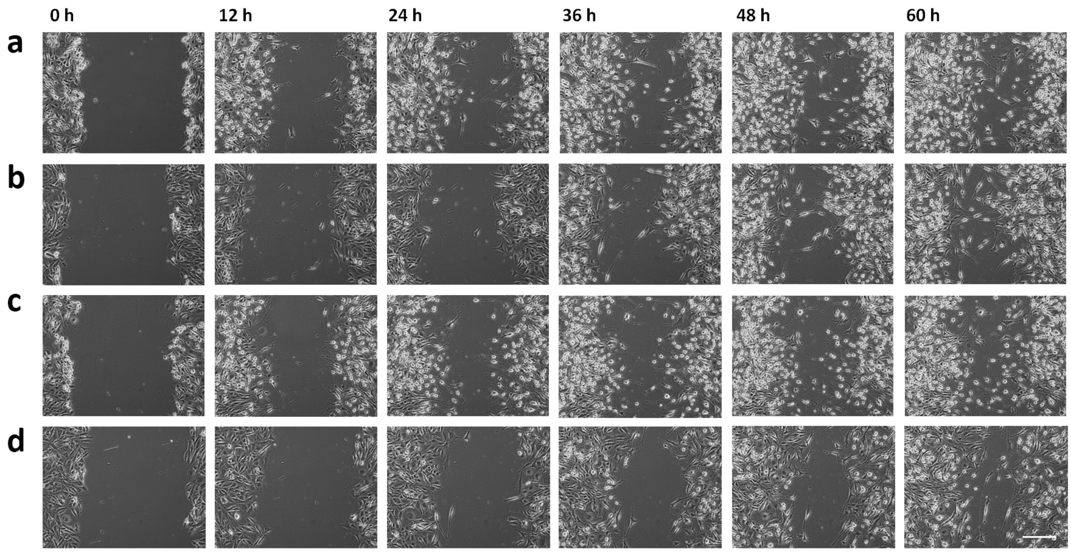

Supplementary Figure 2. Time-lapse images of the migration of HCS-2/8 cells after 0-60 h culture at normoxia in the absence (**a**) and presence (**b**) of 10 μM Y-27632, and at hypoxia (5% oxygen) in the absence (**c**) and presence (**d**) of 10 μM Y-27632.
